# Local coordination state of rare earth in eutectic scintillators for neutron detector applications

**DOI:** 10.1038/srep13332

**Published:** 2015-08-21

**Authors:** Hirokazu Masai, Takayuki Yanagida, Teruyasu Mizoguchi, Toshiaki Ina, Takamichi Miyazaki, Noriaki Kawaguti, Kentaro Fukuda

**Affiliations:** 1 Institute for Chemical Research, Kyoto University, Gokasho, Uji, Kyoto 611-0011, Japan; 2 Kyushu Institute of Technology, 2-4 Hibikino, Wakamatsu-ku, Kitakyushu, Fukuoka, 808-0135, Japan; 3 Institute of Industrial Science, Tokyo University, 4-6-1, Komaba, Meguro, Tokyo 153-8505, Japan; 4 Japan Synchrotron Radiation Research Institute (JASRI/SPring-8), Kouto, Sayo-cho, Hyogo, 679-5198, Japan; 5 Technical Division, Graduate School of Engineering, Tohoku University, 6-6-11, Aoba, Sendai 980-8579, Japan; 6 Tokuyama Corporation, 1-1 Harumi-cho, Shunan, Yamaguchi, 745-0024, Japan

## Abstract

Atomic distribution in phosphors for neutron detection has not been fully elucidated, although their ionization efficiency is strongly dependent on the state of the rare earth in the matrix. In this work, we examine optical properties of Eu-doped 80LiF-20CaF_2_ eutectics for neutron detector applications based on the Eu distribution. At low concentrations, aggregation of Eu cations is observed, whereas homogeneous atomic dispersion in the CaF_2_ layer, to substitute Ca^2+^ ions, is observed in the eutectics at high concentrations. Eu L_III_ edge X-ray absorption fine structure (XAFS) analysis suggests that neutron responses do not depend on the amount of Eu^2+^ ions. However, transparency, which depends on an ordered lamellar structure, is found to be important for a high light yield in neutron detection. The results confirm the effectiveness of the basic idea concerning the separation of radiation absorbers and activators in particle radiation scintillation and present potential for further improvement of novel bulk detectors.

Scintillators, which convert ionizing radiation to thousands of photons, have played a major role in a variety of fields that involve radiation detection including medical imaging, security, astrophysics, particle physics, and searching for natural resources^1^,^2^,^3^,^4^,^5^,^6^,^7^. Among the several types of scintillators, the thermal neutron scintillation detector is one of the most fascinating. The conventional thermal neutron detector is a gas proportional counter filled with ^3^He gas because of high thermal neutron cross-section and the low background γ-ray sensitivity. However, ^3^He gas, which is a product of the decay of ^3^H in nuclear reactors, is no longer available owing to its high demand and declining supply. Therefore, the enhancement of production was impossible^8^,^9^. Meanwhile, there is a demand for sensitive thermal neutron detectors for both scientific and industrial purposes. To resolve this situation, the effort toward the fabrication of novel thermal neutron scintillators to replace the present ^3^He-based systems has been heightened.

To this end, we propose a candidate: ^6^Li-containing solid state materials. Because ^6^Li has a high interaction probability with thermal neutrons based on the ^6^Li(n, α)^3^H reaction with a high Q-value of 4.8 MeV, there are several reports on Li-containing solid state matter exhibiting good scintillation properties of a ^252^Cf neutron source^10^,^11^,^12^,^13^,^14^,^15^. Recently, lithium-containing fluorides have attracted attention regarding the high conversion efficiency attained by low phonon energy. Among these Li-containing fluorides, the activator–doped LiF/CaF_2_ eutectic prepared by a simple solidification method^16^ is reported as a candidate for neutron scintillator applications^17^,^18^. The concept of the scintillation mechanism of rare-earth–doped LiF/CaF_2_ eutectic is based on the separation of the neutron absorber and the conversion of photons by charged particles in the following way. First, neutrons interact with a ^6^LiF layer with thickness of a few microns, and the generated charged secondary particles excite the CaF_2_ layer. Then, the energies of the secondary particles are converted to scintillation photons by the rare earth (activator). For example, it is reported that the figure of merit of Eu-doped LiF/CaF_2_ eutectic scintillators increases with increasing Li concentration, indicating that the high interaction probability of thermal neutrons is suitable for sensitive detection.

On the other hand, the actual distribution of the activator in solid state matter is a critical factor in attaining a high performance from phosphor or light wave convertors, and the dispersion is discussed in several reports using high-resolution transmission electron microscope observations^19^,^20^. However, because of low chemical durability of fluoride against electron beam observation, it is not easy to observe atomic distribution of activator cations in fluorides, and the actual distribution at the atomic level has not yet been clarified. This is especially true for fluorides containing light elements such as lithium as it is a very challenging target to observe. In the present study, we prepare Eu^2+^ activated 80LiF-20CaF_2_ (LiF/CaF_2_) eutectic scintillators, and examine the relationship between structure and ^252^Cf neutron-induced emission properties using a scanning electron microscope (SEM) and a scanning transmission electron microscope (STEM) with a Cs corrector. Based on the obtained results, we have highlighted the possibility for improving the performance of Eu-doped LiF/CaF_2_ eutectics for application in neutron detection.

Figure 1(a) shows optical transmittance spectra of LiF/CaF_2_ eutectics containing different Eu concentrations (0.005, 0.1, and 5 mol%) with the inset graphic showing their appearances. They are visibly translucent or opaque with brownish coloration, and the opacity increases with increasing Eu concentration (also see Supplementary Fig. 1). Because the difference of the refractive index between CaF_2_ and LiF is less than 0.05 (~0.0417), it is expected that the low transparency, even in Eu-free samples, originates from a phase separation with micrometre size regions. Figure 1(b–d) show backscattered SEM images of these Eu-doped LiF/CaF_2_ eutectics. In these figures, brighter parts indicate the existence of heavier elements. Therefore, bright regions are CaF_2_ containing Eu cations, whereas the dark regions are due to LiF. Similar to previous reports^16^,^17^,^18^, a lamella structure with a layer thickness of submicron-order is generated in the eutectics containing small amounts of Eu cations, which is one of the reasons for low transparency. The lamella structure is not oriented along a fixed direction in the sample but is partially oriented in a grain-like region with several hundred micrometres diameter, which is why no periodical absorption is observed in these transmittance spectra. On the other hand, the lamella structure no longer exists in the 1mol%-Eu doped eutectic, and precipitation of large size grains are observed in high Eu concentration samples. The decrease of transparency owing to such irregularity of the structure is shown in Supplementary Figs 2 & 3. It is notable that some aggregation of Eu cations is observed in the LiF/CaF_2_ eutectics with small Eu-doping. With increasing Eu concentration, there seems to be no aggregation spot in the matrix, suggesting that Eu cations are homogeneously dispersed in the CaF_2_ region.

The luminescence spectrum of Eu-doped LiF/CaF_2_ eutectics following neutron irradiation has been unattainable because of power limitations of the neutron radiation source in Japan. However, from X-ray or α–ray induced spectra shown in Supplementary Fig. 4, we assume that a main emission band located around 2.9 eV is attributable to 5d-4f Eu^2+^ emission. This notion is also reported by another paper^21^,^22^. Figure 2 shows ^252^Cf neutron-induced scintillation decay constants τ_1/e_ of Eu-doped LiF/CaF_2_ eutectics as a function of Eu concentration. The inset image shows the neutron-induced scintillation decay curve of the 0.1 mol%Eu-doped eutectic as a typical example. The decay constant of 0.005 mol%Eu-doped LiF/CaF_2_ eutectic is 0.7 μs, assuring that the emission is due to the Eu^2+^ centre. The decay rate by neutron irradiation is almost constant below 0.1 mol% concentration, and the constant increases monotonically with increasing amounts of Eu. This indicates that a significant concentration quenching occurs around 0.1 mol% Eu. The decay constant and the tendency of an inflection point of the decay constants to be located around 0.1 mol% Eu are similar with those of X-ray scintillation decay profiles as shown in Supplementary Fig. 5.

Figure 3(a) depicts ^252^Cf neutron-induced pulse height spectra of Eu-doped LiF/CaF_2_ eutectics along with conventional Li-glass GS20^23^. Quantum efficiencies of the photomultiplier tube (PMT) at 395 nm (emission peak of GS20) and 435 nm (emission peak of Eu:LiF/CaF_2_) are very similar, as they both have levels around 40% when their light yields are directly compared using the value of the multichannel analyser (MCA) channel. In lower Eu-containing samples, up to Eu 0.1 mol%, which seems to be a threshold of the concentration quenching, the light yield exceeds GS20. The distribution of emission centre can be estimated from the energy resolution of the spectra by dividing the half-width half-maximum of the peak by its channel position. The translucent Eu-doped eutectics exhibit a wider distribution compared with transparent GS20, suggesting that the distribution is affected by the transparency of the sample. The distribution also increases with increasing Eu amount. Figure 3(b) shows neutron-induced absolute light yield (photon/neutron) as a function of Eu concentrations. The light yield drastically decreases above 0.1 mol% Eu-doped sample. Here, we focus on the transparency of the sample and discuss the relationship between the transparency and light yield. Both absolute light yield and transparency monotonically decrease with increasing Eu concentration.

Although emission of Eu^2+^ is usually observed by neutron irradiation, the Eu^2+^/Eu^3+^ ratio should be clarified because the starting material is EuF_3_. In order to calculate the Eu^2+^/Eu^3+^ ratio in the total Eu concentration, X-ray absorption fine structure (XAFS) analysis of Eu L_III_ edge is performed. Figure 4(a) shows Eu- L_III_ edge X-ray absorption near edge structure (XANES) spectra of 0.1 Eu- and 5Eu- doped LiF/CaF_2_ eutectics along with the deconvoluted fitting lines. Circles, solid lines, and dashed lines indicate the raw data, fitting curves, and each component, respectively. When comparing the present spectra with previous papers^24^,^25^ and that of EuF_3_ shown in Supplementary Fig. 6, the lower absorption is due to Eu^2+^ whereas the higher one is due to Eu^3+^. Figure 4(b) shows the Eu^2+^/Eu^3+^ ratio and the Eu^2+^ amount of these eutectics as a function of Eu concentration. The Eu^2+^/Eu^3+^ ratio drastically decreases with increasing total Eu concentration, and less than 10% of Eu cations exist as Eu^2+^ in the 5 mol%-doped sample. On the contrary, the amount of Eu^2+^, which is obtained by multiplication of the total Eu concentration and the Eu^2+^/Eu^3+^ ratio, increases. Generally, many secondary electrons (typically several tens of thousands of electrons) in the host are generated by the ^6^Li(n, α)^3^H reaction in the neutron irradiation process due to interactions with charged particles. Therefore, we can conclude that an effective radiative efficiency of the Eu centre is prevented in samples containing higher concentrations of Eu.

As shown in Supplementary Fig. 3(c), it is suggested that an aggregation of Eu species induces concentration quenching. It is often expected that dopant cations segregate at the interface or defect of the sample. Since there is no information about the location of the Eu cations, we have examined the direct observation of the sample using electron beam microscopy. Since segregation of Eu cations possessing high refractive indices will be a source of opacity, it is expected that the dispersion may be a key factor for the fabrication of transparent materials. Figure 5 shows (a) the STEM image of a 5mol% Eu-doped LiF/CaF_2_ eutectics. Energy Dispersive x-ray Spectroscopy (EDS) mapping of (b) F, (c) Ca, and (d) Eu are also shown. Because Li cannot be detected by means of EDS, dispersion of F ions, as shown with dashed lines, indicates the sample shape. As shown in the elemental mapping of the cross-section (dotted lines), the obtained EDS mappings suggest that the Eu cations are incorporated into Ca^2+^ sites. Additionally, apparent aggregation at the interface is not observed, although Eu concentration varies to some extent depending on the observed spot. In the high-resolution high-angle annular dark-field (HAADF) image (e), the Eu cations are observed as a bright spot because of their high atomic number, and the processed image (f) clearly shows that they are regularly dispersed in the crystal lattice of the eutectics. Owing to the lack of aggregation at the edge of the CaF_2_ region, the decrease of emission intensity is explained by concentration quenching, *i.e.* the homogeneous dispersion of Eu cation that induces decrease of the inter-cation distance in the CaF_2_ crystal. The obtained results ensure that the Eu cation located at the CaF_2_ site generates visible photons effectively through energy transfer from the LiF region that absorbs neutron energy.

As in many previous reports^10^,^11^,^12^,^13^,^14^,^15^,^16^,^17^,^18^,^26^, the location of the lanthanide cation in fluoride materials has not been discussed, and only spectroscopic analysis, which is based on the hypothesis that the location is averaged to give similar luminescence intensity, has been performed. The present results suggest that the real valence state and the distribution are important to evaluate the potential of the matrix. For example, small amounts of Eu^2+^ affect a high response to neutron irradiation, although a precise tailoring of the local coordination state is extremely difficult. From this viewpoint, the binary LiF/CaF_2_ eutectic, prepared by spontaneous organization, can possess a hidden potential for improvement, which may be realized with a more precise preparation procedure.

In summary, we have prepared Eu^2+^ activated LiF/CaF_2_ eutectic scintillators by the simple solidification method to investigate neutron-induced luminescence property. In ^252^Cf neutron-induced luminescence spectra, they show Eu^2+^ 5d-4f emission with a decay time of several hundred nanoseconds and the maximum light yield at 0.005 mol% doped Eu. It is expected that the light yield strongly correlates with both the concentration of Eu and transparency of the eutectic. The Eu cations are homogenously dispersed in the CaF_2_ layer without remarkable aggregation. The effective energy transfer from LiF to Eu in the CaF_2_ region suggests that control of the secondary electron is important in addition to that of the nanostructure without degradation of transparency.

## Methods

### Sample preparation

Eu-doped 80LiF-20CaF_2_ (LiF/CaF_2_) eutectics were made by a conventional solidification method using 99.9% pure LiF (^6^Li enriched to 95%), CaF_2_ and EuF_3_ (Stella Chemifa Corporation, Japan). Concentrations of Eu, which was added as a substituent of Ca, were 0.005, 0.02, 0.05, 0.1, 0.3, 1, 3, and 5 mol%. Mixed powders were put into glassy carbon crucible, and the crucible, enclosed by the carbon resist heater, was placed in the stainless steel chamber. After setting up the crucible at the hot zone, the chamber was evacuated up to 10^−4^ Torr. Then, the crucible was heated to 400 °C and kept for about 8 h for baking. After this, the chamber was purged with high purity Ar gas (99.999%) and CF_4_ gas (99.999%) until ambient pressure was reached. The crucible was then heated to 800 °C and kept at that temperature for 30 min. Finally, the furnace was cooled to room temperature with a cooling rate of 5 °C/min. After the fabrication process, the eutectics were cut and polished into pieces of size of 1 × 2 × 5 mm^3^ to investigate optical and scintillation properties.

### Analysis methods

Transmittance spectra were measured using a V670 spectrometer (JASCO). Neutron-induced scintillation responses were evaluated by coupling with a PMT R7600 (Hamamatsu) with an optical grease. The sample was covered with several layers of Teflon tape to collect scintillation photons. The ^252^Cf sealed source (<1MBq), enclosed with a 50-mm-thick polyethylene block as a modulator, was irradiated to the sample. The anode signal from the PMT was fed into a preamplifier (ORTEC 113), a shaping amplifier (ORTEC 570), and an analog-to-digital converter. In addition, neutron-induced scintillation decay time profiles were collected using the oscilloscope (Tektronix TDS3052C).

X-ray excited scintillation emission spectra were investigated using our original system^27^ wherein the supplied voltage of the X-ray tube with a Mo anode was 80 kV, and the tube current was 2.5 mA. In order to compare decay time profiles in photoluminescence and neutron-induced ones, X-ray irradiated scintillation decay time profiles were evaluated by pulse with an X-ray streak camera system^28^ monitoring 435 ± 15 nm.

XAFS measurements of Eu-doped eutectics were conducted at the Eu L_III_-edge at the beam line BL01B1 at SPring-8 (Hyogo, Japan). The operating storage ring energy was 8 GeV with a typical current of 100 mA. The measurements were carried out using a Si (111) double-crystal monochromator in the fluorescence mode using 19-SSD. EuF_3_ pellets, prepared by mixing with BN, were also measured using transmission geometry. Curve fitting of the XANES spectra was performed to determine the Eu^2+^/Eu^3+^ ratio using Athena softwares^29^.

### Microscope observation

A scanning electron microscope (SEM) was used to evaluate the morphology of the sample at the submicron range. SEM observation was performed using a JSM-7100F (Jeol, Japan).

To take the microstructure image with high-resolution and high-sensitivity, we used a scanning transmission electron microscope, STEM (FEI, Titan-Cubed), equipped with a spherical aberration corrector and a monochromator. The acceleration voltage of the microscope was set to 80 kV to decrease knock-on damage. We also applied a low dose rate of 2.53104 electrons/nm^2^/s and a beam blanking system to reduce the total dose. To improve the contrast and resolution of the STEM images, the energy spread of the incident probe was reduced to 0.1 eV in full width at half maximum using the monochromator. STEM images were recorded by a charge-coupled device (CCD) camera. The defocus of the objective lens was set at an underfocus of about 4 nm. Ten STEM images were acquired, each with an exposure time of 2 s, the specimen slightly drifts during the exposure time, but we finally obtained an image with a high resolution by a summation of the ten images.

## Additional Information

**How to cite this article**: Masai, H. *et al.* Local coordination state of rare earth in eutectic scintillators for neutron detector applications. *Sci. Rep.* **5**, 13332; doi: 10.1038/srep13332 (2015).

## Supplementary Material

Supplementary Information

## Figures and Tables

**Figure 1:**
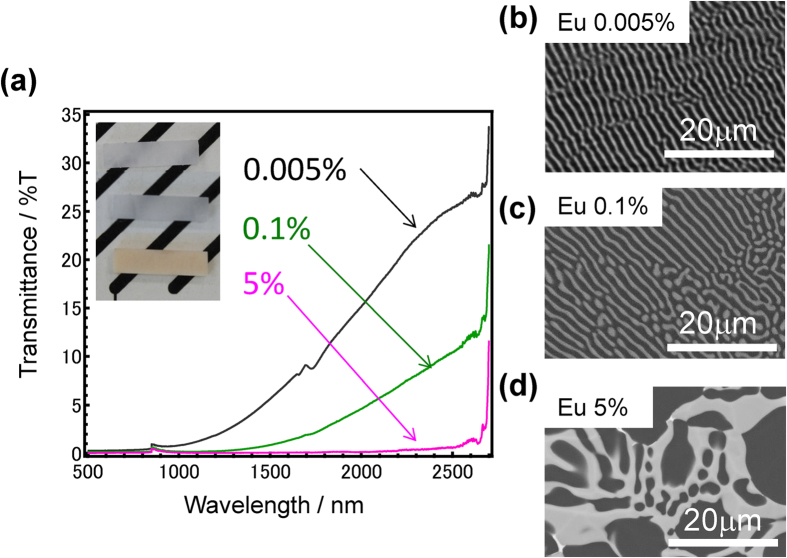
80LiF-20CaF_2_ eutectics containing different Eu concentrations. (**a**) Optical transmittance spectra of several eutectics and the appearances. SEM images of 0.005Eu (**b**), 0.02Eu (**c**), and 5Eu (**d**) -doped samples.

**Figure 2:**
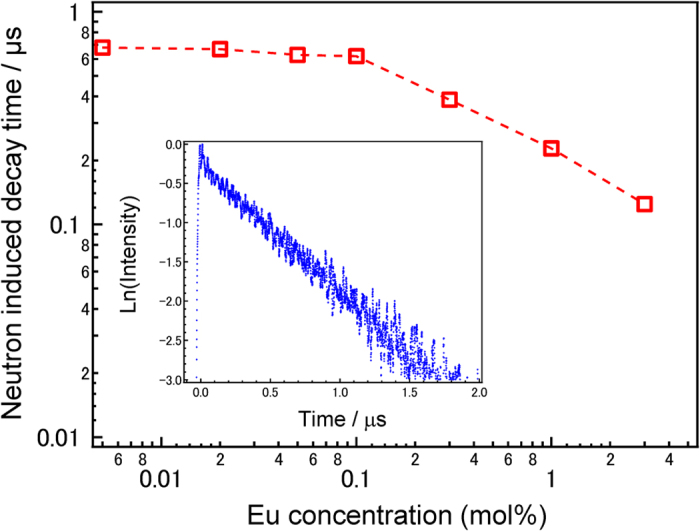
Neutron-induced emission dynamics of Eu-doped 80LiF-20CaF_2_ eutectics. ^252^Cf Neutron excited emission decay curves as a function of Eu concentrations. Inset shows the emission decay curve of the 0.1 mol%Eu-doped eutectic by the neutron irradiation.

**Figure 3:**
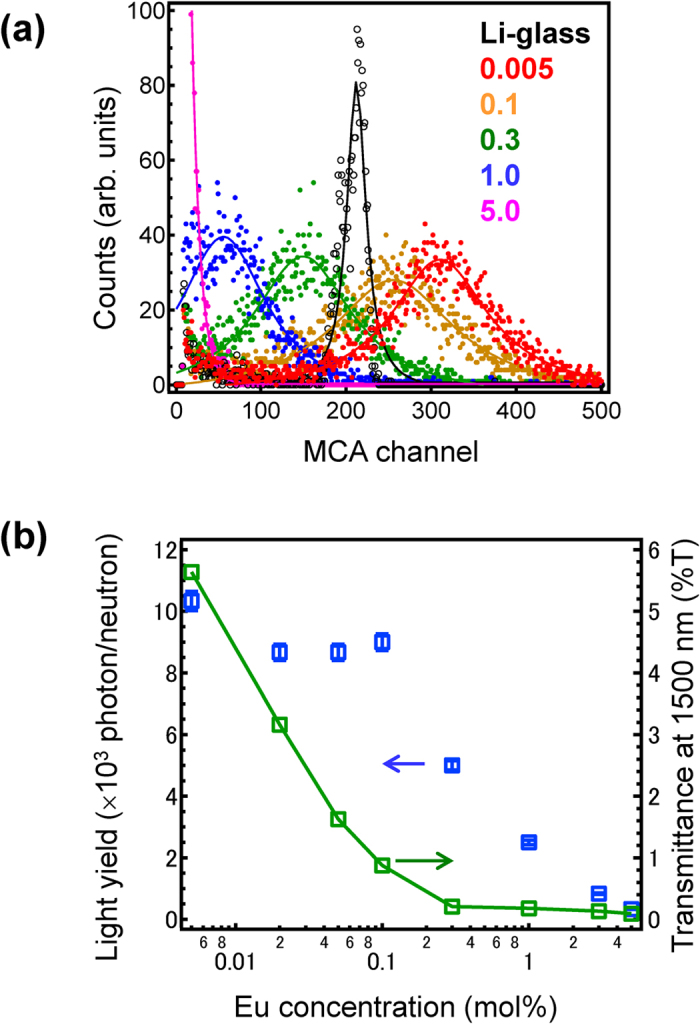
Neutron induced emission of Eu-doped 80LiF-20CaF_2_ eutectics. (**a**) Neutron excited pulse height spectra of LiF/CaF_2_ eutectics along with Li-glass as a reference. (**b**) ^252^Cf neutron excited light yield as a function of Eu concentrations together with that of non-doped sample. The transmittance is also shown as a right axis.

**Figure 4:**
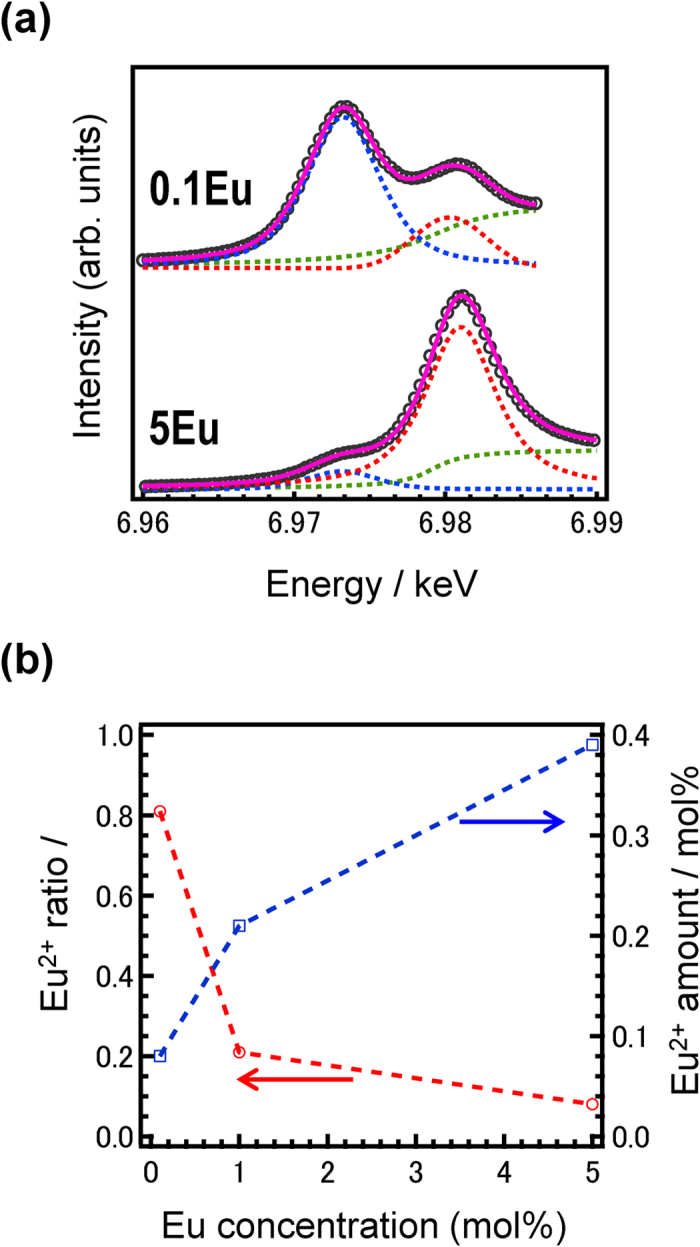
XANES analysis of Eu-doped LiF/CaF_2_ eutectics. (**a**) Eu- L_III_ edge XANES spectra of 0.1Eu- and 5Eu- doped LiF/CaF_2_ eutectics along with the deconvoluted fitting lines. Circles, solid lines, and dashed lines indicate the raw data, fitting curves, and each component, respectively. (**b**) Eu^2+^/Eu^3+^ ratio and Eu^2+^ amount as a function of Eu concentration.

**Figure 5:**
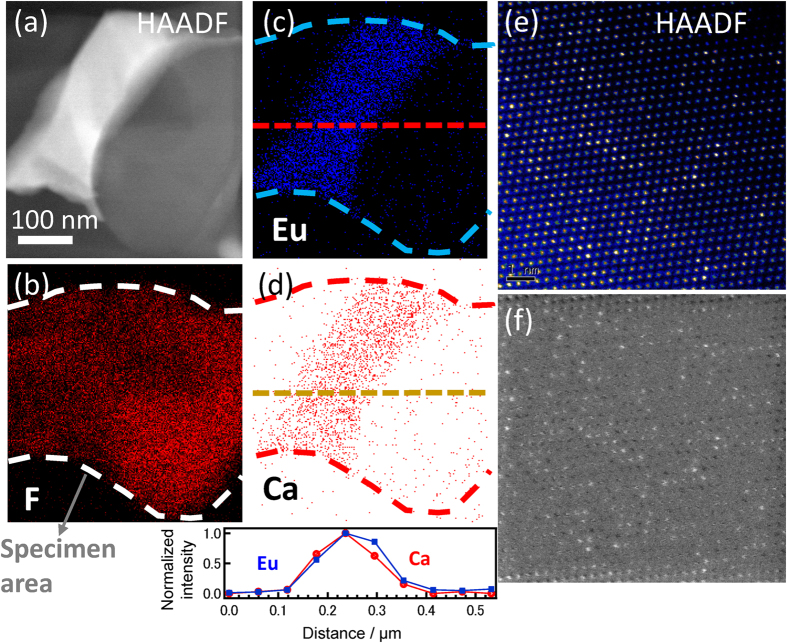
STEM images of 80LiF-15CaF_2_-5EuF_2+δ_ eutectics. STEM image (**a**) and the EDS mappings of F (**b**), Ca (**c**), and Eu (**d**). The elemental mappings of each cation show that Eu cations are homogenously dispersed in the CaF_2_ region without remarkable aggregation at the interface. Bright spots in the HAADF image (**e**) indicate existence of Eu cation in the CaF_2_ at the Ca site. The processed image (**f**) clearly shows that they are regularly dispersed in the crystal lattice of the eutectics.
