# Supplementary material for: Local coordination state of rare earth in eutectic scintillators for neutron detector applications
Source: Sci Rep. 2015 Aug 21;5:13332. doi: 10.1038/srep13332 (PMC4544017; doi:10.1038/srep13332)
Supplement: Supplementary Information [file srep13332-s1.pdf]

Local coordination state of rare earth in eutectic scintillators for neutron detector applications

H. Masai, T. Yanagida, T. Mizoguchi, T. Ina, T. Miyazaki, N. Kawaguti, and K. Fukuda

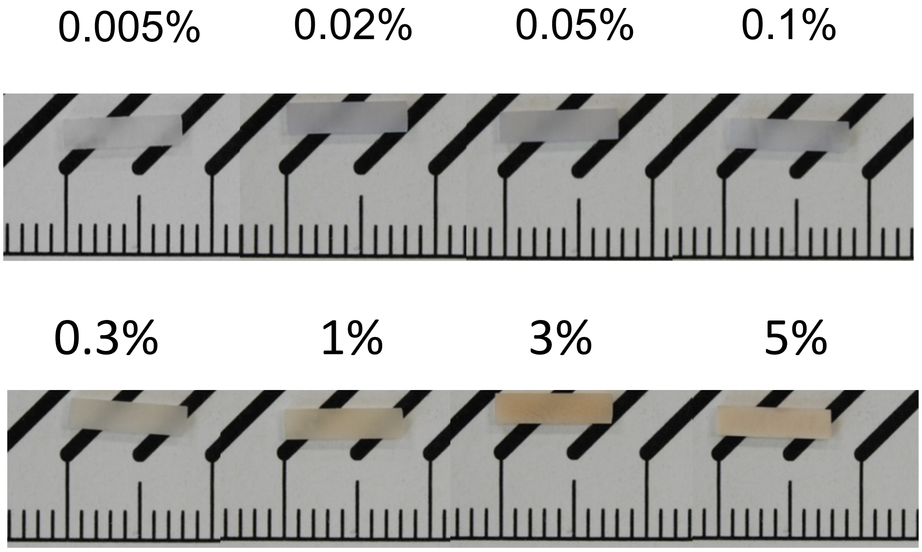

Supplemental Figure 1 | Photograph of LiF/CaF_2 eutectics containing different Eu concentrations.

Local coordination state of rare earth in eutectic scintillators for neutron detector applications

H. Masai, T. Yanagida, T. Mizoguchi, T. Ina, T. Miyazaki, N. Kawaguti, and K. Fukuda

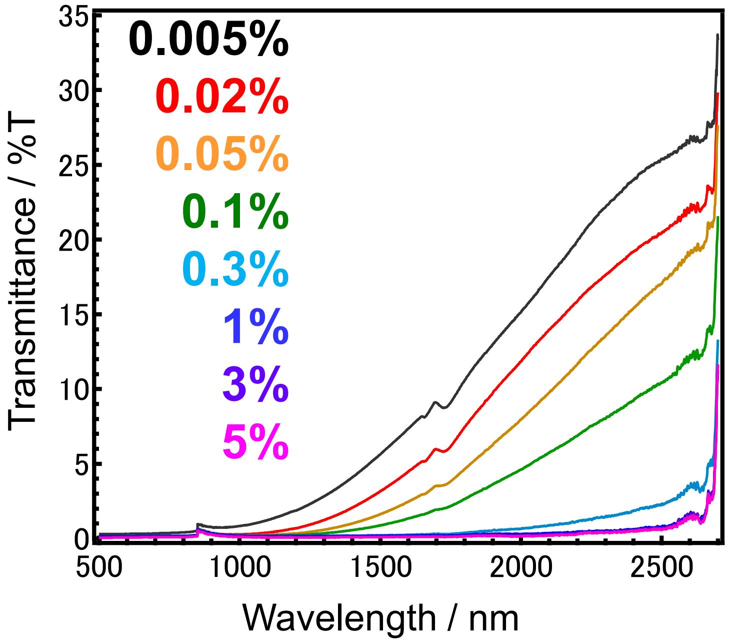

Supplemental Figure 2 | Optical absorption spectra of LiF/CaF_2 eutectics containing different Eu concentrations.

Local coordination state of rare earth in eutectic scintillators for neutron detector applications

H. Masai, T. Yanagida, T. Mizoguchi, T. Ina, T. Miyazaki, N. Kawaguti, and K. Fukuda

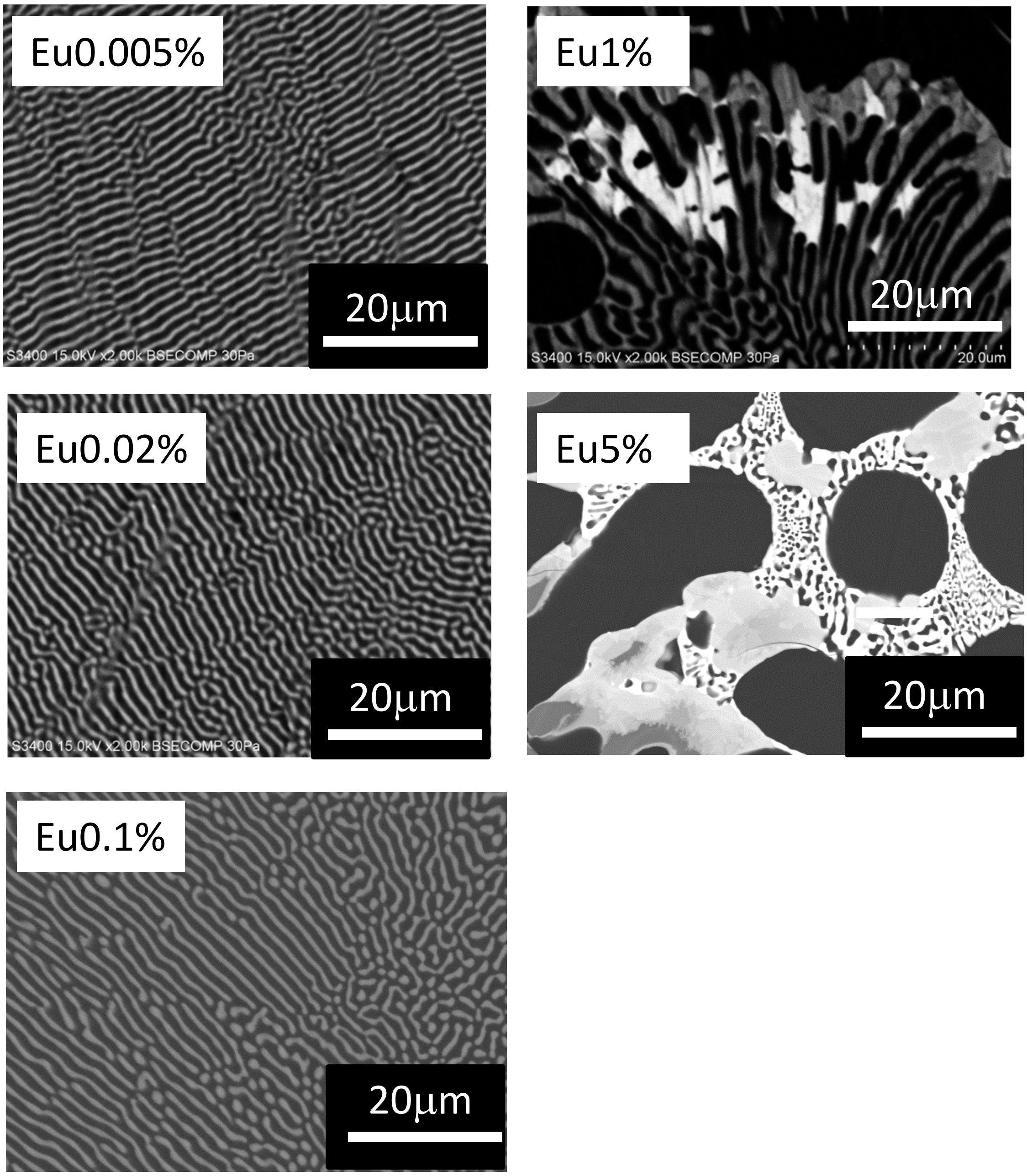

Supplemental Figure 3a | SEM images of LiF/CaF_2 eutectics containing different Eu concentrations.

Local coordination state of rare earth in eutectic scintillators for neutron detector applications

H. Masai, T. Yanagida, T. Mizoguchi, T. Ina, T. Miyazaki, N. Kawaguti, and K. Fukuda

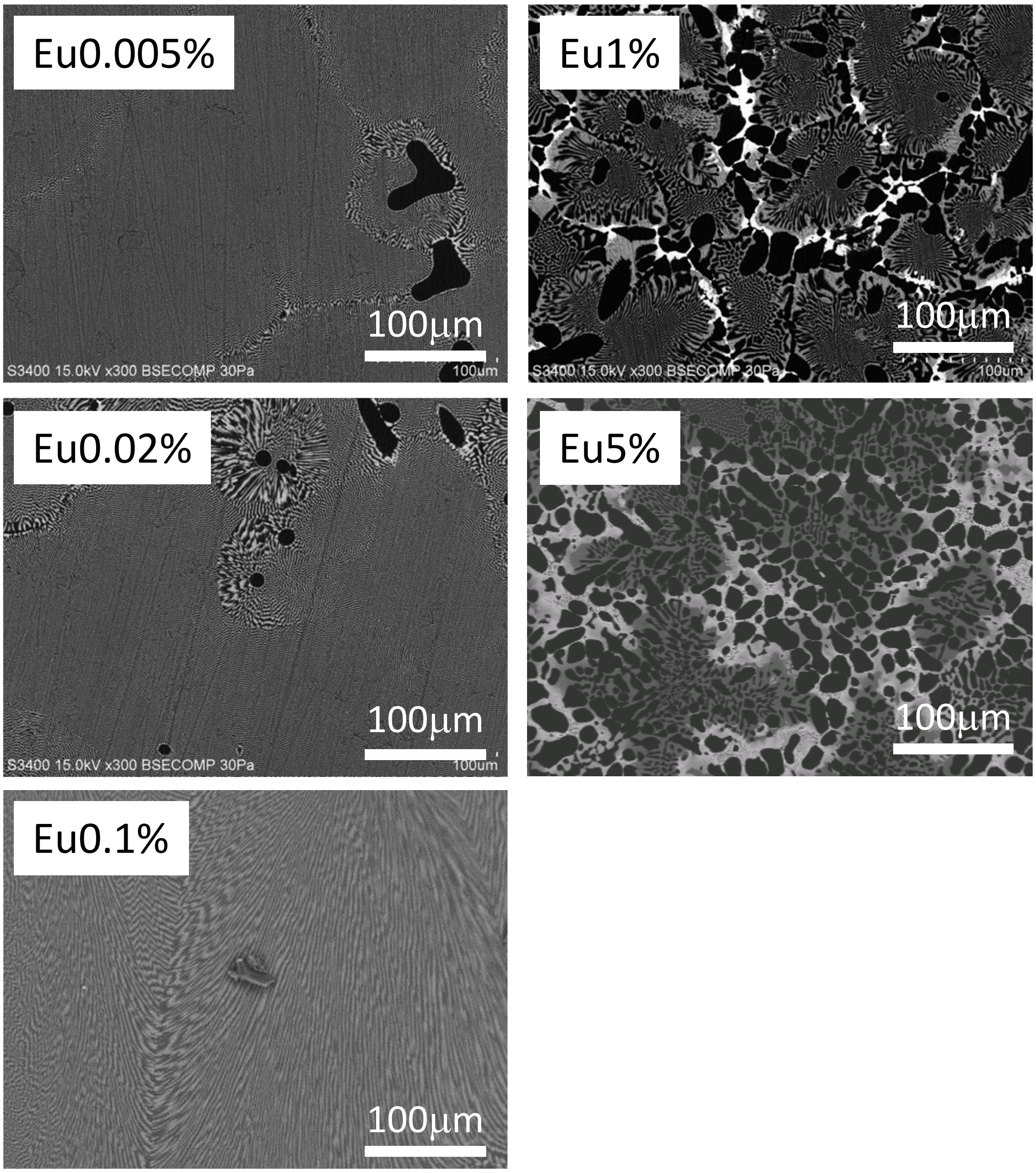

Supplemental Figure 3b | SEM images of LiF/CaF_2 eutectics containing different Eu concentrations.

Local coordination state of rare earth in eutectic scintillators for neutron detector applications

H. Masai, T. Yanagida, T. Mizoguchi, T. Ina, T. Miyazaki, N. Kawaguti, and K. Fukuda

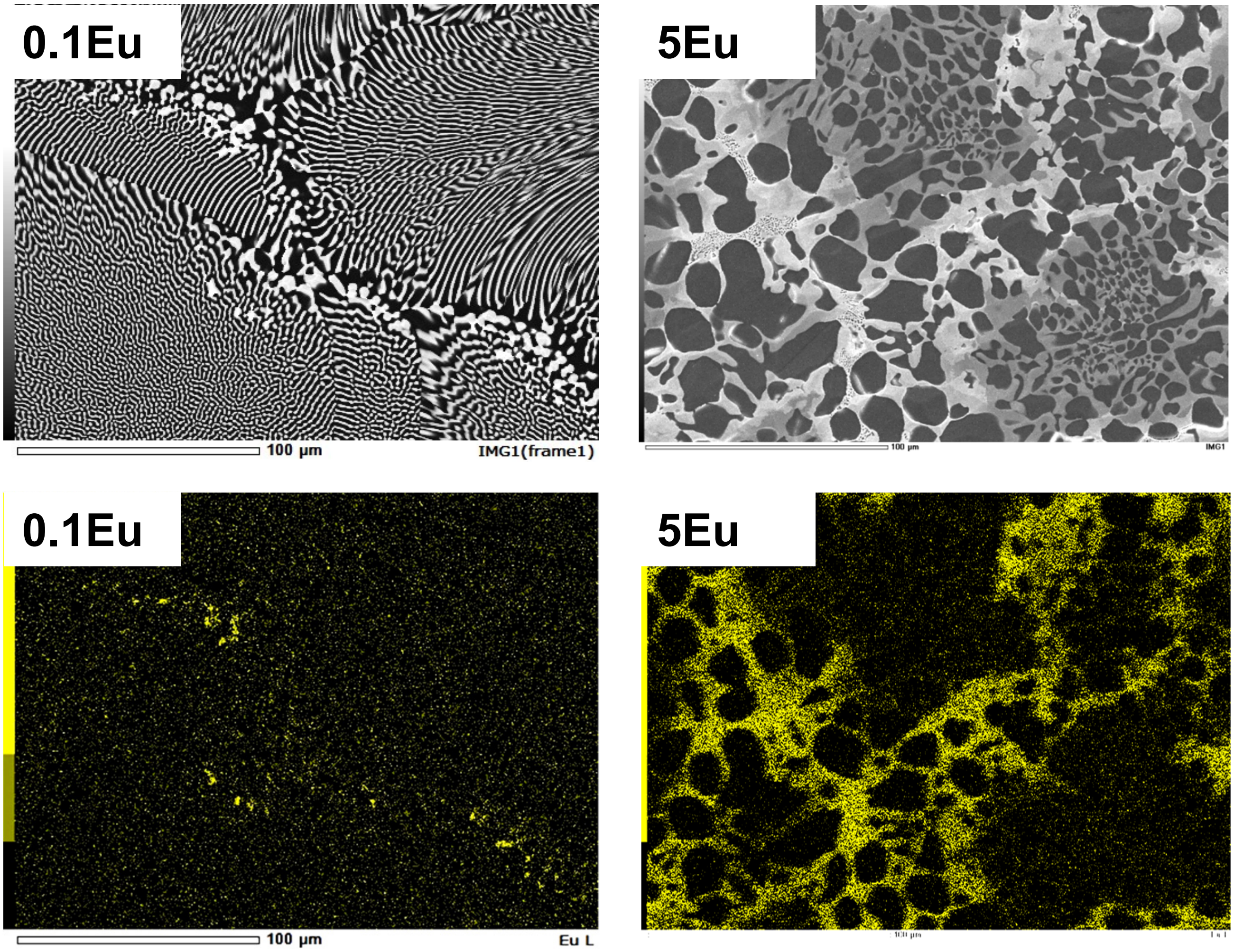

Supplemental Figure 3c | SEM and the EDS images of Eu of 0.1Eu- and 5Eu- doped LiF/CaF_2 eutectics.

Local coordination state of rare earth in eutectic scintillators for neutron detector applications

H. Masai, T. Yanagida, T. Mizoguchi, T. Ina, T. Miyazaki, N. Kawaguti, and K. Fukuda

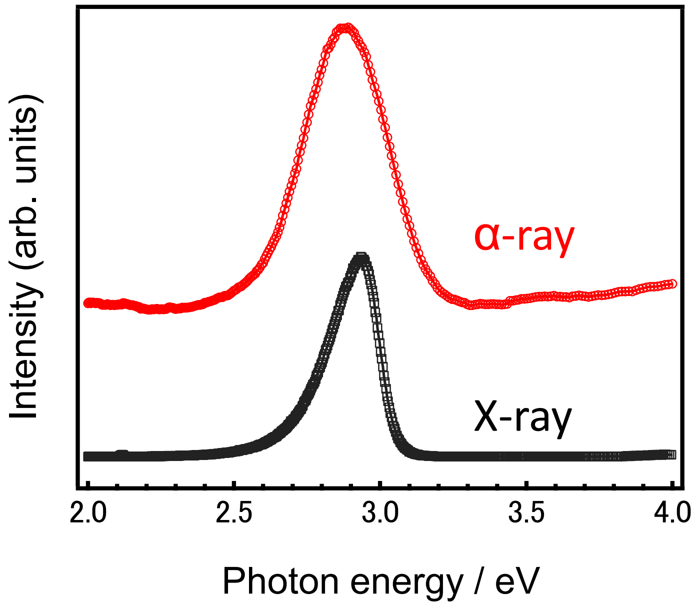

Supplemental Figure 4 | X- and α-ray induced emission of 0.005Eu-doped LiF/CaF_2 eutectics.

Local coordination state of rare earth in eutectic scintillators for neutron detector applications

H. Masai, T. Yanagida, T. Mizoguchi, T. Ina, T. Miyazaki, N. Kawaguti, and K. Fukuda

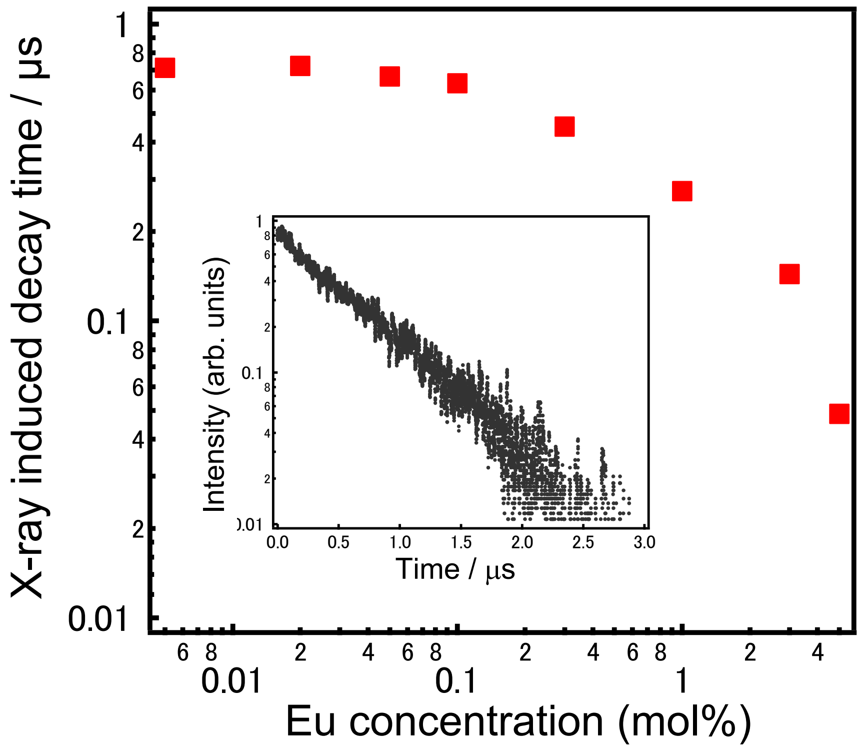

Supplemental Figure 5 | Emission property of LiF/CaF_2 eutectic by X-ray irradiation. X-ray excited emission decay curves as a function of Eu concentrations. Inset shows emission decay curve of the 1.0%Eu-doped eutectic by X-ray irradiation.

Local coordination state of rare earth in eutectic scintillators for neutron detector applications

H. Masai, T. Yanagida, T. Mizoguchi, T. Ina, T. Miyazaki, N. Kawaguti, and K. Fukuda

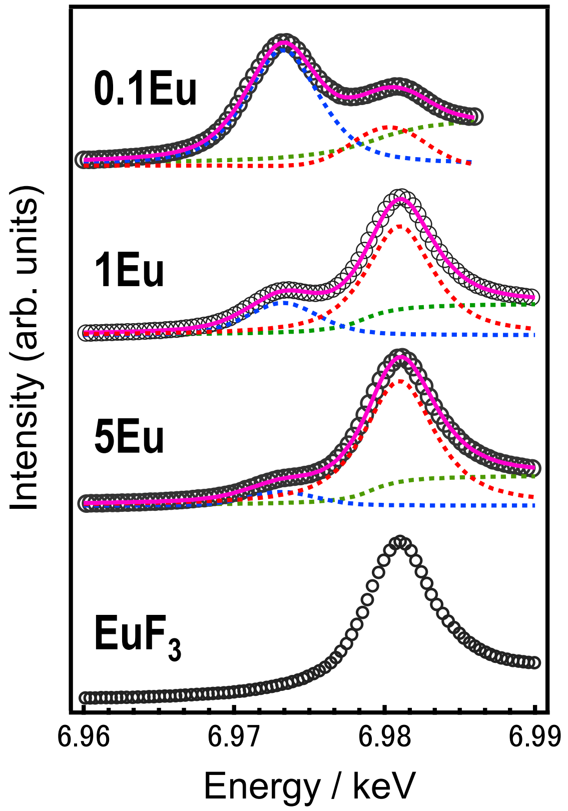

Supplemental Figure 6 | Eu- L_{II} edge XANES spectra of 0.1Eu-, 1Eu-, and 5Eu- doped LiF/CaF_2 eutectics along with that of EuF_3. Dashed lines depict each component after peak deconvolution.
